# Graphene-Based Sensors for Human Health Monitoring

**DOI:** 10.3389/fchem.2019.00399

**Published:** 2019-06-11

**Authors:** Haizhou Huang, Shi Su, Nan Wu, Hao Wan, Shu Wan, Hengchang Bi, Litao Sun

**Affiliations:** ^1^SEU-FEI Nano-Pico Center, Key Lab of MEMS of Ministry of Education, Collaborative Innovation Center for Micro/Nano Fabrication, Device and System, Southeast University, Nanjing, China; ^2^Center for Advanced Materials and Manufacture, Southeast University-Monash University Joint Research Institute, Suzhou, China; ^3^Center for Advanced Carbon Materials, Jiangnan Graphene Research Institute, Southeast University, Changzhou, China

**Keywords:** graphene, sensors, health monitoring, invasive, non-invasive, wearable

## Abstract

Since the desire for real-time human health monitoring as well as seamless human-machine interaction is increasing rapidly, plenty of research efforts have been made to investigate wearable sensors and implantable devices in recent years. As a novel 2D material, graphene has aroused a boom in the field of sensor research around the world due to its advantages in mechanical, thermal, and electrical properties. Numerous graphene-based sensors used for human health monitoring have been reported, including wearable sensors, as well as implantable devices, which can realize the real-time measurement of body temperature, heart rate, pulse oxygenation, respiration rate, blood pressure, blood glucose, electrocardiogram signal, electromyogram signal, and electroencephalograph signal, etc. Herein, as a review of the latest graphene-based sensors for health monitoring, their novel structures, sensing mechanisms, technological innovations, components for sensor systems and potential challenges will be discussed and outlined.

## Introduction

As the global population is growing rapidly and the life expectancy of humans is increasing drastically (Vaupel, 2010; Takei et al., 2015), the healthcare system is facing increasing expenses and burdens, requiring governments to find feasible solutions to render adequate medical care without increasing healthcare costs (Pantelopoulos and Bourbakis, 2010). Preventive and personalized medicine approaches (Ng et al., 2009), which change with health status, can be detected and diagnosed early. Disease-risk can also be predicted, and utilized to overcome challenges by increasing the cure rate and survivability of an at-risk population, while minimizing the overall treatment costs (Narayan and Verma, 2016; Tricoli et al., 2017). By periodically or continuously tracking critical signs and biomarkers, health monitoring systems are capable of comprehensively assessing health conditions which can remarkably benefit the diagnosis and diseases treatment along with postoperative rehabilitation, which can significantly reduce the burden of medical systems and improve quality of life (Yao et al., 2017).

As a critical component of health monitoring systems and the interface to the human body, sensors, including wearable and implantable sensors, are able to detect and measure various signals or analytes with high specificity and sensitivity (Narayan and Verma, 2016). Indeed, due to the mechanical mismatch between the human skin (or soft biological tissues) and conventional rigid silicon-based sensors, mechanical flexibility is notably essential for these invasive or non-invasive sensors (Wang et al., 2017). Moreover, several constraints including biocompatibility, reliability, stability, comfort, convenience, miniaturization, costs, and biofouling should also be considered or even traded for location-unlimited, long-term, multifunctional, real-time, unobtrusive, pervasive, affordable health monitoring (Pantelopoulos and Bourbakis, 2010). Furthermore, recent impressive data management and analysis methods, such as Big Data (Murdoch and Detsky, 2013; Bates et al., 2014; Raghupathi and Raghupathi, 2014), and machine learning (Ravi et al., 2017) technology are applied in data handling and effective information mining (Banaee et al., 2013), since a large amount of data can be collected by these sensors (Someya et al., 2016). Consequently, personal data security and privacy should be effectively guaranteed.

Graphene, owing to its extraordinary multiple properties, such as ultrahigh carrier mobility (Novoselov et al., 2004; Weiss et al., 2012), excellent electrical conductivity, superior thermal conductivity (Balandin et al., 2008; Balandin, 2011), large theoretical specific surface area (Zhu et al., 2010), high optical transmittance (Nair et al., 2008), high Young's modulus (Lee et al., 2008a) and outstanding mechanical flexibility (Yang H. et al., 2018), is a promising 2D material in many applications, especially for the development of wearable sensors and implantable devices in health monitoring. Various and multifunctional sensors can be realized, which benefits from the performance diversities of graphene. The advantages of graphene for sensors are summarized as follows: the first point is that the high specific surface area and the atomic thickness of graphene layers render entire carbon atoms directly in contact with analytes, as a result, graphene-based sensors have superior sensitivity compared to silicon (Justino et al., 2017). In addition, conformal, intimate contact with organs of interest such as the skin (Ameri et al., 2016), brain (Park et al., 2017) and eyes (Kim et al., 2017) can be achieved by graphene-based sensors, because of the mechanical flexibility and ultrathin thickness of graphene, which is essential in acquiring high-quality signals without irritation, motion artifacts, or contamination (Ray et al., 2018). Moreover, high optical transparency and electrical conductivity renders graphene an ideal material for bio-tissue observation with clear images and without visual disturbances (Lee et al., 2015). Furthermore, a high signal-to-noise ratio (SNR) can be achieved in electrophysiological signals recording by the conformal integration and the efficient signal transmission depending on the high electrical conductivity (Ameri et al., 2016). Additionally, the superior performance of graphene in biosensors, such as large specific surface area, convenient functionalization, wide potential window as well as high electron transfer rate, allows receptors such as enzymes, antibodies and deoxyribonucleic acid (DNA) to be efficiently immobilized on the surface of graphene (Szunerits and Boukherroub, 2018). More discussions on the properties, synthesis, characterization, and other applications of graphene and its derivatives have been reported in previous review papers and are not included in this review due to limitations in space (Soldano et al., 2010; Huang M. et al., 2011; Huang X. et al., 2011).

**Figure 1:**
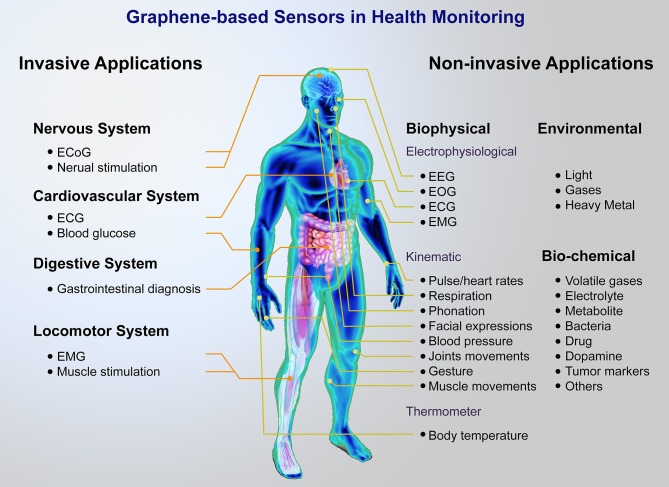
Brief of graphene-based sensor platform for health monitoring. A major distinction can be made between non-invasive and invasive applications, including wearable sensors for monitoring biophysical, biochemical, environment signals, and implantable devices for nervous, cardiovascular, digestive, locomotor system.

As shown in Figure 1, a lot of graphene and its derivatives, including graphene oxide (GO), reduced graphene oxide (rGO), and graphene composites based sensors for human health monitoring have been reported, including wearable sensors, and implantable devices, which can realize the real-time measurement of body temperature (Trung and Lee, 2016; Wang et al., 2018a), heart rate (Karim et al., 2017), wrist pulse (Yang et al., 2017; Pang et al., 2018), respiration rate (Boland et al., 2014; Xu et al., 2018c), blood pressure (Pang et al., 2016), blood glucose (Pu et al., 2018), electrocardiogram (ECG) signal (Ameri et al., 2016), electromyogram (EMG) signal (Yun et al., 2017; Sun et al., 2018) and electroencephalograph (EEG) signal (Ameri et al., 2016; Yun et al., 2017), etc.

In this review, we start from a brief overview about the importance and urgency of health monitoring, as well as the merits of graphene in sensors and its biocompatibility. Following that, we will focus on the latest graphene-based sensors including invasive and non-invasive health monitoring and their novel structures, sensing mechanisms and technological innovations. Components for sensor systems will also be presented. Finally, potential challenges and future prospects of the graphene-based sensing systems are outlined.

## Biocompatibility of Graphene

Although graphene-based sensors have received considerable attention in health monitoring and biomedical applications, it is crucial to consider the impact of graphene and its derivatives on human health such as its biocompatibility, toxicity, as well as its potential risks to the environment before graphene is integrated with human skin, particularly when implanted into the human body. Numerous studies have been devoted to graphene-based nanomaterials (GBNs). However, there is still a lack of systematic research on human health or the environmental effects (Dasari Shareena et al., 2018). Indeed, thorough safety assessments are an essential part of novel materials (Park et al., 2017). It should be noted that the term “grapheme” in research papers generally describe a series of GBNs including GO, and also rGO (Reina et al., 2017; Fadeel et al., 2018). The number of layers, the average lateral size and the carbon-to-oxygen (C/O) ratio are key parameters to classify graphene in different synthesis methods for convenience, due to the lack of standardized descriptions of GBNs (Wick et al., 2014; Reina et al., 2017). The intrinsic physicochemical characteristics of GBNs, such as dose, shape, purity, surface chemistry, layers, thickness, and lateral size, etc., are largely determined by the extent of toxicity and tend to impact their biodistrubution, translocation to secondary organs, accumulation, degradation as well as clearance (Dasari Shareena et al., 2018; Fadeel et al., 2018). The initial properties and biological behaviors of GBNs altered dynamically after exposure to the immune cells, or biomolecules in the biological environment, which may lead to the degradation or biotransformation. Furthermore, characteristics may change when these GBNs move to other biological milieu over time. Therefore, *in-situ* assessments are significant for future applications (Tran et al., 2017). In recent years, the toxicity of GBNs has been evaluated in main target organs including the immune system, cardiovascular system, along with various sorts of organisms such as bacteria, plants, invertebrates, and vertebrates etc. in diversified ecosystems. There is evidence that GBNs can cross physiological barriers and reach secondary organs away from the initial entry. However, it is still too early to draw conclusions owning to the scarcity of data and a lack of understanding regarding the long-term accumulation effects (Fadeel et al., 2018).

Skin, the largest organ of human body and the primary barrier to the environment exposure, is an ideal bio-integrating platform with graphene-based wearable sensors for health monitoring (Liu Y. et al., 2017), and is the most likely location for contact with GBNs. However, the dermal effects of GBNs are still in their infancy and there are few studies available on cutaneous toxicity and toxicological data at present (Pelin et al., 2018). The most likely scenario is the skin irritation and an allergic reaction when there is a cutaneous exposure to GBNs, whereas the tendency of its reaction with proteins cannot be ruled out (Kenry et al., 2016). The cytotoxicity toward skin keratinocytes and fibroblasts *in-vitro* have been investigated in recent studies (Fadeel et al., 2018). One study (Liao et al., 2011) showed that an aggregated graphene sheet had a stronger cytotoxicity to adhere to human skin fibroblasts than reversibly aggregated GO, because of a greater propensity to aggregate. One subsequent study (Pelin et al., 2016) suggested that only high concentrations and a long exposure time to a few layers of graphene (acquired by ball-milling treatment) GO could penetrate human primary keratinocytes as well as harm mitochondrial activity associated with plasma membrane damage, indicating low cytotoxicity toward human keratinocytes together with fibroblasts. Currently an available study (Erf et al., 2017) on dermal effects of GBNs *in-vivo*, where GO was injected into the dermis of a growing feather of a chicken, provided a minimally invasive model for the assessment of the immune response. The result showed that the infiltration of lymphocytes and macrophages increased at the injected position and then decreased gradually. Therefore, the ability to trigger an immune response after dermal injection aroused concerns about GBNs. However, this study is not really an *in-vivo* experiment on the cutaneous dermal effects of GBNs, due to the invasive injection. Overall, with only a few available studies at present, the exact toxicity of GBNs after cutaneous exposure or any conclusions on dermal efforts of GBNs, cannot be sufficiently defined.

The implanted materials should have an excellent biocompatibility and low toxicity with humans, as described. Thus, the safety assessment of graphene-based implants is of predominate significance. Since graphene-based implantable sensors are currently mainly utilized in neuroscience, herein we emphasize the effects of GBNs to the central nervous system. Graphene is an attractive material for the implementation of multifunctional brain implantable devices, owing to the unique physicochemical properties including the flexibility, high optical transparency and electrical conductivity. However, the brain cells and neuronal circuits are directly exposed to graphene-based implants (Fadeel et al., 2018).

Early studies (Li et al., 2011; Bendali et al., 2013; Sahni et al., 2013; Tu et al., 2014) have shown that neural cells cultured on planar graphene/positively charged GO surfaces, can survive with remarkable viability, normal neuronal metabolism and morphology, even enhanced adhesion, or improved neurite sprouting, outgrowth as well as branching. However, more recent studies (Bramini et al., 2016; Rauti et al., 2016) have presented that lateral size-related graphene/GO flakes impact on neuronal transmission and network functionality despite having no effect on the cell viability and network formation. While no primary neurons and glial cell death occurs in the long-term exposure to graphene or GO in explanted studies, it has a lateral size-dependent impact on several fundamental physiological processes, which may potentially cause toxicity for chronic exposure. In fact, the restoration of pathological changes in the central nervous system can be exploited by utilizing some characteristics of GBNs. Furthermore, despite *in vitro* studies with rich data, the impact of GBNs *in-vivo* on neuronal microcircuits is still lacking. Longstanding assessments are crucial to confirm the biocompatibility and overall safety of the graphene-based neural implants (Kostarelos et al., 2017).

In summary, the current studies on biocompatibility of GBNs are still controversial on account of the high heterogeneity of GBNs on the market and various synthesis methods (Bramini et al., 2018). It should be noted that GBNs may produce a varying extent of potential toxicity to cells associated with a direct interaction with the cell membrane (Syama and Mohanan, 2016). So far, GO is preferred to original graphene for biomedical application, due to its surface chemistry, better solubility and stability in biological fluids (Bramini et al., 2018). Hence, future studies should fine-tune the properties of functionalizing GBNs to acquire selected performance, while avoiding its potentially adverse effects if possible.

To overcome these limitations and to extend the biological applications of GBNs, many graphene-biomacromolecule hybrid materials, such as graphene-biopolymer nanohybrids (achieved by combining GBNs with biocompatible polymers), graphene-polysaccharide nanohybrids (achieved by combining GBNs with biocompatible polysaccharides), have been synthesized to meet the demands of biomedical and pharmaceutical application with enhanced biocompatibility, minimized toxicity, improved solubility as well as stability and even to promote cell proliferation (Liu T.C. et al., 2016; Li et al., 2017). Moreover, a number of green routes have also been proposed to reduce the toxicity in the fabrication of rGO with the utilization of microbes, plant extracts and reducing sugars such as glucose instead of strong chemical reducing agents (Syama and Mohanan, 2016). Additionally, avoiding direct contact between GBNs and the human body and even biological fluids, is also a potential method.

In addition to the biological toxicity and biodegradability, material biodegradability should also be considered and ensured in the event of exfoliation or tear when designing implantable devices (Kostarelos et al., 2017). Furthermore, the antibacterial activity of GBNs have also been highlighted in tissue engineering, which have certain antibacterial or antimicrobial properties and can decrease the threat of bacteria, as GO can be adsorbed by the bacterial cells and its sharp edges can damage the cell membrane, which results in cell damage or death (Liu T.C. et al., 2016; Li et al., 2017). The intracellular oxygen partial pressure is also altered by GO, which results in oxidative damage of the intracellular substances, destruction of the cell internal composition and ultimately cell death. As the most effective antibacterial agents among GBNs, GO and rGO are deemed as a superb material for the synthesis of innovative antibacterial agents (Liu et al., 2011).

## Current Applications

Two main areas for graphene-based sensors are currently being pursued: non-invasive (wearable) sensors and invasive (implantable) devices. This section provides an overview of diversified applications of graphene-based sensors, both through non-invasive and invasive means for health monitoring.

### Non-invasive Sensors

Non-invasive health monitoring sensors, typically wearable sensors and electronic artificial skin (e-skin), which do not tend to infiltrate and break in the skin or tissue when detecting vital signals and biomarkers, include a wide range of biosensors for biophysical, biochemical, and environmental signals. This section summarizes the graphene-based non-invasive sensors that graphene and its derivatives provide as the foundation of these systems, through the discussion of a number of the most significant and latest reported sensors for continuous, real-time monitoring of critical parameters for human health.

#### Biophysical Signals

##### Electrophysiological measurement

The active cells or tissues (such as human body and animal tissues), produce regular electrical phenomena, whether in a static state or an active state. This regular electrical phenomenon is known a bioelectrical signal. As for the mechanism of bioelectric signals, it is predominantly a transmembrane flow of ions, which includes a resting potential (RP) and action potential (AP). For static potential, a potential difference between the inside and outside of the cell membrane, is a discrepancy led by an uneven distribution of sodium and potassium ions on both sides of the cell membrane. The different cells' RP is disparate: nerve cell (−86 mV), ventricular myocyte (-90 to 80 mV), purkinje fiber (−100 to 90 mV), sinus node cell (−70 to 40 mV). The active potential, is produced when the cell is stimulated and excited from the outside. A series of transient changes will occur in the membrane potential at the stimulated site, with an initial increase in membrane potential followed by a gradual recovery to resting potential. Clinical bioelectric signals can commonly be gathered by electrodes and becomes an electrocardiogram (ECG), electroencephalogram (EEG), electromyography (EMG), electroretinogram (EOG) after appropriate treatment such as amplification, filtering and post-treatment.

As a vital electrical signal representing the state of human body, it has been utilized in various fields including mobile health care, cognitive psychology and human-machine interactions (Evenson et al., 2015; Steinhubl et al., 2015). However, due to their common mode noise, feeble value and high contact impedance, accurate signal acquisition becomes a prerequisite for signal analysis. Hence, the quality of the electrodes plays a momentous role in measuring the bioelectrical signal. Indeed, bioelectrical electrode sensors should have a broad dynamic range, accuracy, high SNR, low impedance, robustness, durability, and outstanding repeatability among deformation ranges. Nevertheless, existing bio-electrodes are strained by various aspects, i.e., it is expensive to manufacture and has inferior contact with skin. This leads to no guarantee of a stable signal acquisition when a human is in kinetic state. In other words, this limits the real-time collection of bioelectrical signals. With the development of the wearable biometric sensors, there are plentiful and innovate solutions for real-time, high fidelity, low impedance, as well as high SNR for bioelectrical signal acquisition.

In order to obtain accurate, reliable, real-time bioelectric signals, bioelectric electrodes should have excellent mechanical and electrical properties. All the properties of graphene and its derivatives meet the performance requirements of biological electrodes. For instance, graphene is the thinnest conductive medium, it is mechanically robust, biocompatible, and electrochemically stable (Lee et al., 2008b; Nair et al., 2008; Geim, 2009; Pinto et al., 2013; Ameri et al., 2014, 2016). In addition, Graphene and its derivatives are convenient easily shaped into varying types of structures, and this property enhances the practical value of graphene.

**Figure 2:**
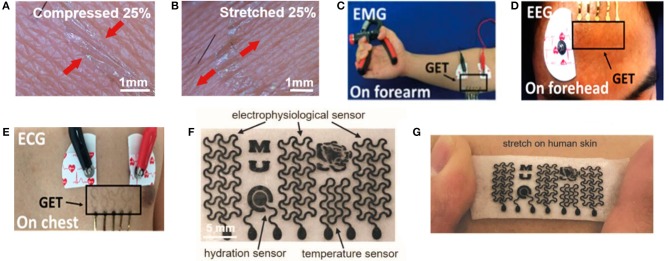
Schematic diagram and its test diagram of graphene-based bioelectrical electrode. Magnified photographs of a GET on compressed **(A)** and stretched **(B)** skin. **(C)** EMG sensing on the forearm with the GET and gel electrodes. **(D)** EEG sensing on the forehead with both the GET and gel electrodes. **(E)** ECG measured synchronously by the GET and gel electrodes. Adapted with permission from Kabiri Ameri et al. (2017). **(F)** Optical images of the as-made on-skin bioelectronics sensing systems **(G)** Sensor mounting on skin. Adapted with permission from Sun et al. (2018).

Graphene-based bioelectrical electrodes with different composite materials and structures have been achieved efficiently through various types of electrodes, as shown in Figure 2. These bioelectrical electrodes have significant merits including excellent mechanical properties, flexibility, outstanding electrical properties, low contact resistance, and high SNR. Since the conventional tattoo-like epidermal sensors were made of thin metal films, and silicon membrane, Kabiri Ameri et al. (2017) proposed a cost- and time-effective method called “wet transfer, dry patterning” to fabricate the graphene electronic tattoo (GET). This innovative sensor had a thickness of 463 ± 30 nm, a stretchability of more than 40%, excellent adhesion with skin via just van der Waals forces and permeability with the open-mesh structure. Later, the GET sensor was successfully suitable for obtaining the various bioelectrical signals containing ECG, EEG, EMG (Figures 2A–E). Because the epidermal devices were fabricated using high-cost methods, which included intricate vacuum microfabrication processes, this peculiarity-imposed restrictions on the widespread adoption of wearable electronics. Yun et al. (2017) proposed a low-cost, solution-based method employing rGO and porous polydimethylsiloxane (PDMS) to fabricate high-performance bioelectrodes. These graphene bioelectrodes had super str*etc*hability with a maximum strain of 150%, excellent durability up to 5,000 cycles of compression and a low sheet resistance of about 1.5 kΩ per square, which showed the potential for low-cost processing and rage-scale application for future wearable electronic skin. Concerning comfort, the most up-to-date electronic devices are made of materials that limit its gas permeability. This characteristic constrained perspiration evaporation and thus brought sequentially adverse physiological and psychological effects. In addition, for widespread use, the device fabrication process should not contain E-beam or photolithography, etching, thin-film deposition or other complex procedures. Sun et al. (2018) proposed multifunctional on-skin electrodes in which the laser-patterned porous graphene plays the role of an active component, while a sugar-templated silicone elastomer sponge plays the role of a substratum. This multipurpose electrode exhibited high water-vapor permeability (18 mg cm^−2^.h^−1^), high water-wicking rates (1 cm/30 s). With such characters, the devices would exhibit excellent air permeability and minimize the inflammation risks to improve long-term feasibility (Figures 2F,G). All these biophysical signals can be measured by graphene-based bioelectrical electrodes including EEG, ECG, EOG, EMG. In essence, their strategy principles are the same.

##### Kinematic detection

In recent years, numerous efforts have been made to develop flexible and stretchable sensors for human motion detecting and monitoring. The dynamic motions and physical activities at different sites of the human body could generate a wide range of crucial signals from daily exercise monitoring to clinical diagnosis, such as movement disorders, respiratory disorders, blood pressure and athletic performance tracking (Trung et al., 2015), which could be captured by highly sensitive pressure, tactile or strain sensors with conformal integration for continuous monitoring. Various structures and sensing materials are utilized to achieve flexible, highly sensitive pressure, tactile, and strain sensors. Diverse sensing mechanisms, such as piezoresistive, piezocapacitive, piezoelectric, piezophototronic and triboelectric types, are employed for skin-integrated flexible sensors, according to different functional materials. Among these sensing mechanisms piezoresistive and piezocapacitive designs that measure the resistance and capacitance changes, respectively, are generally used owing to their facile designs and direct data acquisition (Ray et al., 2018). This emphasizes the advances made in highly sensitive pressure, tactile and strain sensors, using graphene as a sensing material.

The pressure generated by human dynamic motions and physical activities can be divided into three different pressure regimes: a low-pressure regime (< ~10 kPa, such as gentle touch), medium-pressure regime (10–100 kPa, such as heart rate, blood pressure wave) and a high-pressure regime (>100 kPa, such as sole pressure caused by body weight) (Mannsfeld et al., 2010; Lu et al., 2012), among which the medium-pressure regime has been developed most. Indeed, pressure sensors should have a broad dynamic range, high sensitivity, linearity, rapid response, robustness, durability, and good repeatability, among which sensitivity and pressure ranges are the two most important parameters (Huang et al., 2018; Yang H. et al., 2018). The gauge factor (GF), the relative change in resistance (Δ*R*/*R*_0_) divided by applied force (ε), is a significant metric of sensor performance (Lu et al., 2012). Furthermore, several trade-offs are mostly required in sensor designs, such as sensitivity, mechanical stretchability, high and wide working linearity (Pang et al., 2018; Ray et al., 2018; Xia et al., 2018).

Graphene and its derivatives have been commonly used in pressure sensors because of their significant piezoresistive performance and easy handling into varied structures (Yang H. et al., 2018). In recent years, graphene-based flexible pressure sensors with different composite materials and structures have been achieved through their excellent sensing properties (Lv et al., 2017). Diverse methods that use different conduction mechanisms, such as a millefeuille-like architecture, lotus-leaf-like hierarchical structures, graphene elastomer, nanowires/graphene heterostructures, spinosum microstructure, foam, and sparkling blocks have been suggested for heartbeat/wrist pulse monitoring, radial artery monitoring, respiration, phonation/acoustic waves, walking states, finger bending, wrist blood pressure and facial expressions, etc. (Pang et al., 2016, 2018; Chen Z. et al., 2017; Coskun et al., 2017; Huang et al., 2018; Kou et al., 2018; Shi et al., 2018). These methods will contribute to the increased sensitivity of these pressure sensors. However, their cumbersome fabrication and high production costs may limit their applications to some extent.

**Figure 3:**
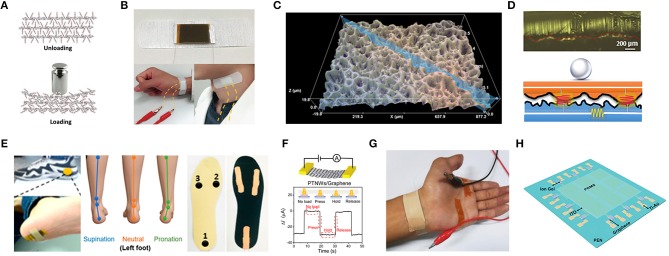
The mechanism and application of flexible graphene-based pressure sensors. **(A)** Schematic illustration of the inner structure change of the functionalized graphene upon loading pressure. **(B)** Image of radial artery pulse and carotid artery pulse detection assembled by adhesive bandage. Adapted with permission from Huang et al. (2018). **(C)** 3D morphology of the graphene pressure sensor using abrasive paper. **(D)** Photograph and schematic illustration of circuit model corresponding to light loading. **(E)** Photograph of the sensor put on the heel of the foot (left) and illustration of foot states for supination, neutral, pronation (middle) and photograph of pressure sensors fixed on the insole (right). Adapted with permission from Pang et al. (2018). **(F)** Pressure response of a PTNWs/G transistor under a pressure pulse. **(G)** Photograph of the measurement for wrist pulses. Adapted with permission from Chen Z. et al. (2017). **(H)** Schematic description of a 3 × 3 graphene tribotronic array. Adapted with permission from Khan et al. (2016).

Recently, several piezoresistive graphene-based pressure sensors with different composite materials and architectures have been developed, which have significant merits including high sensitivity, a broad detection range, low power consumption and facile signal read-out (Chen S. et al., 2017). Since the conventional planar structure piezoresistive pressure sensors suffer from poor sensitivity and are unable to detect in low-pressure regimes, an available approach is based on the employment of micro-structured, micro-patterned and porous-structured sensing materials. Huang et al. (2018) proposed an innovative approach to fine-tune and enhance the sensitivity of pressure sensors by utilizing a millefeuille-like multilayer architecture of rGO intercalated by covalently tethered molecular pillars as a sensing material, which had low cost production, facile fabrication, low operating voltage (0.2 V) and compatibility with printed electronics solutions (Figures 3A,B). This pressure sensor exhibited a sensitivity of 0.82 kPa^−1^, response time (24 ms), low detection limit (7 Pa), high durability (over 2,000 times) and robustness, which can be implemented to monitor wrist pulse and carotid artery pulse for an arterial stiffness diagnosis. Additionally, bioinspired by a high-performance force sensing structure of the epidermis tissue in human skin, Pang et al. (2018), developed a pressure sensor with a random height distribution spinosum microstructure using a simple and low-cost fabrication process, which employed abrasive paper as a template due to the similar topography to epidermis and graphene as a sensing material. Since the effective interlocking of random distribution spinosum layers and sharp morphology, a more homogeneous pressure distribution was achieved compared to the other regular morphologies including the pyramid and hemisphere, which led to a sensitivity of 25.1 kPa^−1^ in a linear range of 0–2.6 kPa. This pressure sensor can be employed to detect the wrist pulse and walking states, monitor the heart rate and respiration states and recognize the voice and phonation signals (Figures 3C–E).

However, several micro-structure graphene-based piezoresistive pressure sensors may suffer from cumbersome and expensive fabrication processes, as an alternative, a piezoelectric pressure sensor is applied. Graphene has presented a significant potential for nano-electromechanical systems such as the negative piezoconductive effect of mono-layer graphene (Huang X. et al., 2011; Chen Z. et al., 2017). The piezoelectric effect, which alters mechanical energy into electrical signals due to the occurrence of electrical dipole moments when mechanical force is applied, has been applied in pressure sensors for sensing dynamic signals and shows a great potential in implementing self-powered sensors. Nevertheless, a number of limitations exist for the piezoelectric pressure sensors including performance degradation, unreliable static response, and a pyroelectric effect which is a confounding factor in sensing. Moreover, piezoelectric sensors are not usually chosen because of their lower pressure sensitivities (Mannsfeld et al., 2010; Huang et al., 2018). Since these piezoelectric-induced pressure sensors face challenges in measuring static signals because the output voltage generated by the piezoelectric materials is an impulsive signal, which could only be observed when driven by the piezopotential from dynamic stress, Chen S. et al. (2017), proposed a nanowires/graphene heterostructures piezoelectric pressure sensor for static measurements with mechanisms that strain-induced polarization charges in piezoelectric nanowires, which can work as charged impurities and impact the carrier mobility in graphene, which is employed an uncomplicated fabrication method. This piezoelectric pressure sensor with enhanced sensitivity (9.4 × 10^−3^ kPa^−1^), a fast response time (5–7 ms), was applied to monitor the radial artery of a human (Figures 3F,G).

Tribotronics has also recently been utilized for pressure sensors, which the charges transport in a FET, coupled with the extrinsic stimulation through triboelectrification (Chen Z. et al., 2017). Graphene with ambipolar transport behaviors is a desirable material for the fabrication of triboelectric nanogenerators (TENG), since the carrier transport can be modulated by either of the triboelectric potential (positive or negative) due to contact electrification between two materials. Khan et al. (2016), proposed a graphene tribotronic touch sensor consisting of a chemical vapor deposition (CVD) graphene FET and a single-electrode-mode TENG integrated in a coplanar fashion. This sensor exhibited a touch-sensing performance with a detection limit (< 1 kPa), a sensitivity of 2% kPa^−1^, a response time of 30 ms, and low power consumption (180 μW), which provided a great significance for e-skins (Figure 3H).

A piezocapacitive sensing mechanism is another prevalent path for pressure sensors due to their higher sensitivity, lower hysteresis, and lower power consumption (Chen S. et al., 2017; Ray et al., 2018). However, by miniaturizing the sensing unit of the capacitive pressure sensors, the effect of parasitic capacitance is increased when it is close to the capacitance of each unit and more susceptible to the ambient, which leads to the low SNR. Because of its high flexibility, excellent electrical conductivity, and large surface area, graphene and its conductive elastomers have been employed as both the dielectric materials and electrodes (Wan et al., 2017). Kou et al. (2018) proposed a flexible capacitive pressure sensor that consisted of graphene/polydimethylsiloxane (Gr/PDMS) dielectric layer, PDMS substrate, the wrinkled Au electrode and antenna, which showed the sensitivity of 0.24 kPa^−1^ in the low-pressure regime (0–10 kPa) and 0.0078 kPa^−1^ in the high-pressure regime (10–100 kPa). This sensor also exhibited a low detection limit of 5 Pa, and a response time of 67 ms, which could be employed to detect subtle pressures such as facial expressions and hand bending.

Flexible strain sensors, which measure the deformation of objects (Lu et al., 2012), should have a desirable performance, such as a high sensitivity, superb stretchability, stability, durability, linearity, fast response, and high SNR among other criteria. Among these features, sensitivity and stretchability that are determined by the threshold sensing level and working range without damage, respectively, are the two significant parameters that commonly require a trade-off between them. It is essential to maintain conductivity for the sensing material in order to achieve a wide range of strain (Xu et al., 2018c). As described previously, GF is also a significant metric of strain sensor performance. Graphene can be employed as piezoresistive strain sensors, not only due to its excellent strain sensitivity, and mechanical properties, but also changes in the electronic band structure and the resistance of graphene. Indeed, the hexagonal structure near the edge of the graphene film would partially be damaged when tensile strain is applied (Zang, 2013). Thus, many demonstrations have been presented on graphene-based piezoresistive strain sensors. However, the perfect graphene for strain sensing has a low sensitivity owing to its sensing mechanism that the zero bandgap can be opened under high tensile strain (23% for uniaxial) (Nair et al., 2008). For increasing the sensitivity of graphene-based strain sensors, several structural innovations have been developed recently, such as using aerogel, graphene textile, fiber yarns, porous structure/foam, fish-scale-like structures, woven fabrics, films, spring like mesh, grid-patterns, graphene/glycerol-KCl networks, liquid forms, piezopotential-gated coplanar graphene transistors, gels, as well as mazelike networks, etc., for pulse/heart rate/blood pressure monitoring, vocal cord vibration/swallowing/acoustic wave, human body motion monitoring including gesture recognition, joint bending, facial expressions, muscle movements, etc. (Lu et al., 2012; Zang, 2013; Sun et al., 2015; An et al., 2016; Liu Q. et al., 2016; Lou et al., 2016; Cai et al., 2017; Lee et al., 2017; Wan et al., 2017, 2018; Kou et al., 2018; Liu C. et al., 2018; Ma et al., 2018; Shi et al., 2018; Souri and Bhattacharyya, 2018; Wu et al., 2018; Xu et al., 2018a,b,c; Yang Z. et al., 2018; Yuan et al., 2018; Yu et al., 2018; Zhang et al., 2018). The presence of these graphene-based strain sensors indicate a promising solution to home health monitoring and point-of-care testing (POCT) devices.

**Figure 4:**
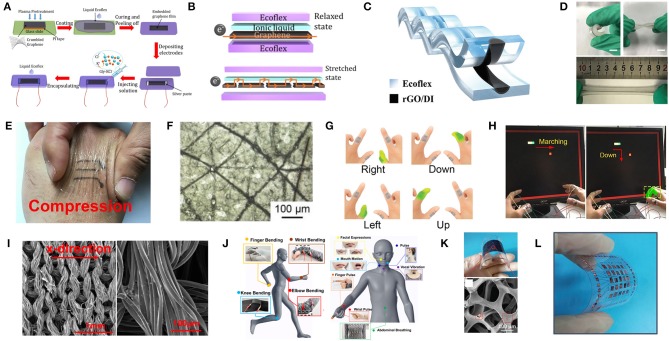
The preparation, mechanism, and application of flexible graphene-based strain sensors. **(A)** Schematic of process for fabricating the Gly-KCl based strain sensor. **(B)** Schematics illustrating the electron pathway in the strain sensor in the initial state and with applied strain. Adapted with permission from Liu C. et al. (2018). **(C)** Schematic of the cross-section of the strain sensor based on rGO/DI sensing liquids and Ecoflex rubber. **(D)** Photographs of the strain sensor under bending, torqueing and stretching. Adapted with permission from Xu et al. (2018c). **(E)** Photograph of the nose film on skin with graphene pattern under compression. **(F)** Optical microscopy images of the substrate. **(G)** The set of commands consisting of bending different fingers for human-machine interaction. **(H)** Photograph of playing a video game with the movement of the bar being controlled by appropriately bending the corresponding finger. Adapted with permission from Wan et al. (2018). **(I)** SEM image of the graphene textile and entire wire in the x-direction. **(J)** Detection of various human motions in different sensing locations using the wearable graphene textile strain sensor. Adapted with permission from Yang Z. et al. (2018). **(K)** Photograph of the bended strain sensor and SEM image of 3DGF/CNTs percolation networks. **(L)** Photograph of the 5 × 5 array of the 3DGF/CNT networked strain sensor. Adapted with permission from Cai et al. (2017).

The majority of flexible strain sensors are based on nano-sheets or network structures as sensing elements. Therefore, Liu C. et al. (2018) developed a liquid-state-based wearable strain sensor with a graphene/glycerol/potassium chloride (graphene/Gly-KCl) ionic conductor as the sensing element and Ecoflex as the encapsulant. As a deformable Gly-KCl ionic conductor combined with cracked graphene sheets in conductive path, this sensor showed a stretchability of 300%, a gauge factor of 25.2, and a response time of 80 ms, which could be utilized to monitor large-scale and subtle human motions including joint movement, facial expressions and pulses (Figures 4A,B). Additionally, Xu et al. (2018c) reported a novel highly stretchable and durable strain sensor based on rGO/DI (deionized water) sensing liquids and Ecoflex rubber with a facile, low-cost and scalable process, with rGO foams mixed with DI and packaged by rubber. The sensing mechanism was based on reversible micro-contact among rGO nano-foams in the sensing fluid, which led to the resistance changes when stretching or compressing deformations were applied. This strain sensor showed both stretching and compressing properties (maximum GF of 31.6 and pressure sensitivity of 0.122 kPa^−1^), a sensing range up to 400% in strain and 87 kPa in pressure, a low limit of detection (0.1% strain), superb reliability and stability (>15,000 cycles for pressuring and >10,000 cycles for stretching), distinguishing capability between compression, tensile deformation and reusability. This sensor could be utilized to monitor varied human-motions such as drinking, phonation, wrist bending and fist clenching, along with distinguishing stretching signals from pressing signals (Figures 4C,D). Since human skin is relatively rough with dermatoglyph, although tape-fixation may work to some extent, it is challenging to gain an ultra-conformal contact with skin. For this purpose, Wan et al. (2018) developed an ultra-conformal, biodegradable strain sensor with a simple, low-cost, double transfer process based on a commercial make-up accessory (nose film) and graphene as the substrate and active materials, respectively. The sensing mechanism is based on the sliding of the nanosheets in fish-scale like graphene layers under an applied strain. This sensor showed a strong adhesion (a peel-off strength 29.4 N.m^−1^), a high sensitivity (gauge factor, GF: 502), a rapid response (54 ms), as well as a skin-level stretchability (35%), which could monitor the vital physiological signals including vocal cord movements, jugular venous pulses and radial artery waves, enabling human-machine interaction (Figures 4E–H). Yang Z. et al. (2018) fabricated a close-fitting and wearable graphene textile strain sensor with the employment of thermally reduced GO dyed polyester fabric, which had a negative resistance when the applied strain was increased. In addition, this sensor exhibited a high sensitivity (GF = −26, strain range 8%, y-direction/GF = −1.7, strain range 15%, x-direction), high stability and comfort, which could be incorporated into clothing to monitor subtle and large human motions (Figures 4I,J). Cai et al. (2017) developed an all-carbon nanoarchitecture strain sensor based on 3D graphene foam and carbon nanotubes (3D-Graphene/CNTs), which showed a GF of 35, high stretchability (up to 85%), excellent SNR, and which could be easily mounted on human skin for real-time monitoring of human motions and even for acoustic vibration recognition (Figures 4K,L). A summary of graphene-based pressure and strain sensors is presented in Table 1.

**Table 1:** Graphene-based pressure sensors and strain sensors.

**Sensing materials**	**Type**^**§**^	**Sensing mechanism**^**†**^	**Detecting range (kPa/%)**	**Sensitivity (kPa^**−1**^)/GF**	**Response time (ms)**	**Detection limit (Pa)**	**References**
Mille-feuille assembly of rGO	P	PR.	0–0.6	0.82	24	7	Huang et al., 2018
Graphene/PDMS film	P	PR.	0–25	1.2	/	5	Shi et al., 2018
Spinosum microstructure rGO	P	PR.	0–2.6	25.1	120	≤16	Pang et al., 2018
Graphene porous network/PDMS	P/S	PR.	0–1	0.09 25.6	100 /	/	Pang et al., 2016
Air–bubbled graphene block	P	PR.	0–0.12	229.8	/	/	Lv et al., 2017
MXene/rGO aerogel	P	PR.	1–3.5	22.56	< 200	10	Ma et al., 2018
3D Graphene/PDMS hollow structure	P	PR.	0–60	15.9	1.2	/	Luo et al., 2017
Wrinkled graphene film/PVA nanowires	P	PR.	3–10	28.34	87	2.24	Liu W. et al., 2018
Fingerprint–like 3D graphene film	P	PR.	0–0.2	110	< 30	0.2	Xia et al., 2018
rGO paper	P	PR.	0–20	17.2	120	/	Tao et al., 2017
CNTs/graphene/PDMS	P	PR.	< 0.3	19.8	16.7	0.6	Jian et al., 2017
Graphene/GO composite film	P	PR.	0–900	0.0002–0.0012	/	/	Liu S. et al., 2017
Graphene/porous Al_x_O_y_ membrane	P	PR.	0.3–1.5 1.5–4.5	6.92 0.14	/	~300	Chen W. et al., 2017
rGO/PVDF–(TrFe) nanofibers	P	PR.	20–60	15.6	5	1.2	Lou et al., 2016
Vanadium nitride-graphene	P	PR.	2–10	40	130	/	Yu et al., 2018
Micro–patterned graphene/PDMS	P	PC.	0–10 10–100	0.24 0.0078	< 100	5	Kou et al., 2018
GO foam	P	PC.	0–1	0.8	100	0.24	Wan et al., 2017
PbTiO_3_ nanowires/graphene	P	PE.	0–1.4	9.4 × 10^−3^	5–7	/	Chen Z. et al., 2017
Graphene FET/triboelectric	P	TE.	0–10	0.02	30	< 1	Khan et al., 2016
Graphene/polyester fabric	S	R.	x–axis: 0–15%	GF = −1.7	/	/	Yang Z. et al., 2018
			y–axis: 0–8%	GF = −26			
Fiber/GNP/carbon black	S	R.	0–60%	GF = 1.46–5.62	209	/	Souri and Bhattacharyya, 2018
Porous rGO film	S	R.	0.25–0.75%	GF = 282.2	/	/	Xu et al., 2018b
3D graphene foam/CNTs	S	R.	0–85%	GF = 35	30	/	Cai et al., 2017
Graphene/nose film	S	R.	0.8–35%	GF = 502	54	/	Wan et al., 2018
Fish–scale–like rGO film	S	R.	0–82%	GF = 16.2–150	/	0.1%	Liu Q. et al., 2016
Graphene woven fabrics	S	R.	0–2%	GF = 500	/	/	Yang et al., 2017
			2–6%	GF = 10^3^			
			>8%	GF = 10^6^			
rGO/acetylcellulose film	S	R.	0.17–0.43%	GF = 208	150	/	Xu et al., 2018a
				GF = 495			
Graphene/polymer springlike mesh	P/S	R.	80 0–110%	72 GF = 0.37	/	1.38 0.1%	Yuan et al., 2018
rGO/deionized water	P/S	R.	87 0–400%	0.122 GF = 31.6	60.3	/	Xu et al., 2018c
						0.1%	
Grid–patterned graphene nanoplatelet	S	R.	5%, 10%−25%	GF = 301.61–29631	100	/	Lee et al., 2017
Graphene/Glycerol–KCl solution	S	R.	0–300%	GF = 25.2	80	/	Liu C. et al., 2018
graphene /PVDF–(TrFE) transistors	S	PE	0–0.12% 0.12–0.3%	GF = 389 GF = 69	/	0.008%	Sun et al., 2015
Graphene/PAAM/PVP/ethylene glycol (EG) organogel	S	R.	0–9,200% 9,200–10,500%	GF = 0.8 GF = 2.3	160	/	Zhang et al., 2018
Mazelike vertical graphene film	S	PR.	120% 55%	GF = 32.6 GF = 88.4	960	/	Wu et al., 2018

§ *P, pressure; S, strain*;

† *PR, piezoresistive; PC, piezocapacitive; PE, piezoelectric; TE, triboelectric; R, resistive*.

##### Thermometer

Warm-blooded animals are a long-term evolution of cold-blooded animals, while human beings are typical representatives. From a physiological point of view, monitoring human body temperature is of great significance as it reflects the body's metabolic level. For sick and injured patients, body temperature can be introduced to assess recovery rate. For athletes, it can provide a reference for their training and competition arrangements (Sahatiya et al., 2016). Usually people only measure body temperature when they are unwell. However, continuous body temperature monitoring is receiving increased attention. For instance, body temperature is related to our biological clock and can be utilized to research human being's sleeping patterns. To meet the requirements above, temperature sensors should have biocompatibility, high thermal responsivity, stability, reproducibility, linearity, and if possible, optical transparency, stretchability and mechanical flexibility (Trung et al., 2014). Usually the principle of a resistive temperature sensor is the resistance of sensitive material changes with temperature alteration, as the relationship between resistance and temperature is the sensitivity (Wang et al., 2018a). To obtain more precise data and to maintain timely measurements, a real-time monitoring technique is required. Consequently, wearable solutions have been proposed.

The thermal properties of graphene, such as thermal conductivity, has captured the attention of many researchers. Compared to metals and carbon nanotubes, graphene has a higher thermal conductivity which is promising in thermal applications and energy storage fields. With the techniques of fabricating complex graphene structures in microscales and nanoscales, graphene is becoming an excellent candidate for temperature sensors due to its great electronic properties, remarkable mechanical strength and high thermal conductivity (Davaji et al., 2017). In recent years, various graphene-based temperature sensors emerged, which attracted widespread attention. Trung et al. (2018) proposed wearable temperature sensors fabricated by freestanding single reduction graphene oxide (rGO), of which the resistance is dependent on temperature. These sensors do solve many problems like real-time monitoring, stretchability, transparency, thermal conductivity and so on, however, the complex fabrication steps and high cost limit its widespread use.

Recently, several graphene composite sensors with different materials have been reported, which have a stable sensing performance under deformation of the sensor, high sensitivity and long duration. Wang et al. (2018a) developed a stretchable temperature sensor consisting of a cellular graphene/PDMS composite. The desired structure of the sensor was fabricated using 3-D printing technology, which forms long-range ordered and precisely controlled cellular microstructures, including grid, triangular, and hexagonal porous structures.

With these porous structures to share the external strain, the composites show better sensitivity than solid composites. For instance, the grid structure shows only a 15% sensitivity decrease at a large tensile strain of 20%. As the next generation of wearable devices may need to be integrated into clothes, Trung et al. (2018) developed fiber-based wearable temperature sensors using freestanding single reduction graphene oxide (rGO) fiber. In addition, the thermal index was tunable by using wet spinning and by controlling the GO's reduction time. This sensor has a fast response time (7 s), good recovery time (20 s) and high responsivity to temperature. When a deformation occurs, the response will be maintained. As the sensor is fiber-based, it can be conveniently integrated into socks or undershirts and can monitor body temperature in real time.

#### Biochemical Signals

Although measuring biophysical signals offers a window into the health status of human body, there are a lot of limitations for a comprehensive assessment, which commonly requires further considerations of biochemical signals. In addition, traditional biochemical measurements utilize costly biochemical analytical instruments with trained personnel in centralized laboratory facilities, which involve sampling (a biofluid, commonly blood), pretreatment, and further analysis by professional instruments for identifying and quantifying the concentrations of biochemical markers of interest (Ray et al., 2018). Moreover, conventional testing is typically invasive, expensive, complicated and time consuming. Accordingly, there are increasing requirements in cost-effective, continuous, non-invasive, real-time, portable wearable biochemical sensing devices for fast, point-of-care detection to address these constraints.

Biochemical sensors are extremely promising for wearable health monitoring owing to their high specificity, rapidity, portability, low price and power consumption. The primary sensing mechanisms of a classic biochemical sensor are as follows: a receptor such as an enzyme, antibody, DNA or whole cell is employed for specific recognition of the target analyte in the samples and generates physicochemical signals. Then the transducer, such as an electrochemical, optical and mechanical transducer translates the signal into an electrical, optical signal that can be quantified (Kim et al., 2019). Currently available biochemical sensors on the market, such as blood glucose test strips, have been widely applied for blood analysis and require blood sampling through an invasive, painful process, especially for infants, the elderly and diabetics, which also carries the potential risk of infection or being unsuitable for high sampling rates in continuous monitoring. As an alternative to blood, biofluids such as sweat, saliva, tears and interstitial fluid (ISF), can be readily sampled in a non-invasive, user-friendly manner without breaking the protecting layer of the skin and contacting blood, which contain a wealth of health-related biochemical targets and show the potential for health monitoring. For instance, the concentration of chloride, lactate and glucose in sweat can be utilized, respectively, to screen for cystic fibrosis in infants, identify the beginning of pressure-induced ischemia and the transition between an aerobic and anaerobic state during physical activities (Ray et al., 2018), as well as detect blood glycemic transitions for diabetes management (Kim et al., 2019). Wearable biochemical sensors render an approach to non-invasive, continuous, real-time, routine monitoring of biomarkers in these biofluids for the management of chronic diseases and for monitoring abnormal and unforeseen situations. However, challenges exist before practical utilization can occur, including a deep understanding of the analyte correlations, proportionality between biofluids and blood biochemical composition, physiology of biofluid secretion, physiological variance among individuals, lag, scope, validation, stability, accuracy, sample volumes, secretion rates, filtration, active analyte channels, variable pH and salinity, analyte breakdown, and storage, etc. (Heikenfeld et al., 2019). Although there has been rapid progress in wearable biochemical sensors technology in recent years, commercially successful applications remain elusive for biochemical analytes beyond glucose, which are still in their infancy for wearable biochemical sensors to improve the performance of sensors and quality of life. Additionally, the majority of electrochemical biosensors are utilized *in vitro* to detect the analytes in metabolites, blood or artificial serum, therefore, biosensors for real-time measurements *in-vivo* should be a priority in the future.

The superior performance of graphene in biochemical sensors, such as large specific surface area, facile modification, wide potential window, high electron transfer rate, high charge-carrier mobility and low electrical noise levels, allows highly sensitive detection, efficient receptor immobilization, easy interaction with biomolecules, promotion of electron transfer between the reagents and graphene, etc. (Justino et al., 2017; Szunerits and Boukherroub, 2018). Compared with conventional carbon electrode based sensors, graphene-based biochemical sensors show better performance, including sensitivity, limits of detection (LOD) and response time. Thus, a number of graphene-based biochemical sensors have been demonstrated in health monitoring, including the detection of electrolytes (i.e., sodium, potassium and calcium, etc.) and metabolites (i.e., lactate and glucose, etc.) in biofluids such as sweat, ISF, saliva or tears, the monitoring of volatile organic compounds (VOCs) in the exhaled breath (i.e., acetone, ethanol, and ammonia) and other biochemical targets (i.e., heavy metals, ketamine, bacteria, and neurotransmitter, etc.). The flexible graphene-based non-invasive sensors for detection of biomarkers in biofluids are shown in Table 2.

**Table 2:** Graphene-based flexible non-invasive biochemical sensors^†^.

**Sensing materials**	**Analyte sample**		**Wearable platform/Sensing Mechanism**	**Sensitivity**	**Detection range**	**Detection limit**	**References**
Graphene/GOx/CAT	Glucose in tears		Contact lens/ElectroChem	22.72%/mM	0.1~0.9 mM	12.57 μM	Park J. et al., 2018
Graphene/GOx	Glucose in tears		Contact lens/ElectroChem	/	1 μM~ 10 mM	1 μM	Kim et al., 2017
Prussian blue/gold-doped graphene-hybrid/GOx	Glucose in sweat		Patch/ElectroChem	/	10 μM~ 0.7 mM	10 μM	Lee et al., 2016
Pt nanoparticles-graphene hybrid/hydrogel/GOx	Glucose in ISF		Patch/ElectroChem	37 μA mM^−1^.cm^−2^	6 μM~ 0.7 mM	0.76 μM	Lipani et al., 2018
PtAuNP/ rGO/CHIT/GOx	Glucose in sweat		Patch/ElectroChem	48 μA mM^−1^.cm^−2^	0~2.4 mM	5 μM	Xuan et al., 2018b
PtAuNP/3D porous LIG/AgNW/GOx	Glucose in sweat		Patch/ElectroChem	6.4 μA mM^−1^.cm^−2^	0~1.1 mM	5 μM	Xuan et al., 2018a
Au nanowrinkles/rGO/polyurethane	Glucose in sweat		Patch/ElectroChem	140 μA mM^−1^.cm^−2^	1 μM ~1 mM	500 nM	Toi et al., 2019
Electroreduced graphene oxide/cortisol and lactate antibodies	Cortisol	In sweat/saliva	Patch/ElectroChem	/	0.1~200 ng.mL^−1^	0.1 ng.mL^−1^	Tuteja et al., 2018
	Lactate				0.5~25 mM	0.1 mM	
Amino-functionalized graphene paper/Cu_3_(btc)_2_ nanocubes	Lactate	In sweat	Patch/ElectroChem	29 μA mM^−1^.cm^−2^	0.05~22.6 mM	5 μM	Wang et al., 2018b
	Glucose			5.36 mA mM^−1^.cm^−2^	0.05 ~1.776 μM	30 nM	
Au and Co_3_O_4_ modified SGGTs/GOx/CHIT and SGGTs/BSA/CHIT	Glucose	In tears	As prepared/ElectroChem	/		100 nM	Xiong et al., 2018
	Uric acid						
AOx/graphene platelets/PS-*b*-PAA	Ascorbic acid	In tear film	Strip/ElectroChem	/	5~350 μM	4.93 ± 0.53 ng/μL	Khan et al., 2017
		In aqueous humor				39.63 ± 4.4 ng/μL	
Graphene/AMP	Bacteria in saliva		Patch/LC resonant	/	/	Single bacterium	Mannoor et al., 2012

† *GOx, glucose oxidase; ElectroChem, electrochemistry measurement; PtAuNP, platinum and gold nanoparticles; LIG, laser-induced graphene; SGGTs, solution-gated graphene transistors; CHIT, chitosan; BSA, bovine serum albumin; AOx, ascorbate oxidase; PS-b-PAA, poly(styrene)-block-poly-(acrylic acid); AMP, antimicrobial peptides*.

##### Metabolites

Analytes in blood are separated from ISF, saliva, sweat and tears by thin, cell-based barriers, and the ease and route of diffusion are related to the morphology and composition of these barriers, which result in differences in composition. For small molecules (for example, Na^+^, K^+^, glucose, and lactate), the ISF concentration is extremely similar to the plasma concentration due to the rapid paracellular diffusion through the capillary walls. On the contrary, for large molecules (such as proteins and lipids), the analyte concentrations in ISF is diluted compare to blood, which are relaxed to molecular weight. However, for saliva and sweat, the paracellular route results in substantial dilution of most analytes. If analyte concentrations change rapidly in the plasma, there is a time delay before the corresponding change occurs in the ISF, saliva, sweat and tears. Although these biofluids contain rich targeted analytes of interest, currently, the majority of studies are focused on monitoring glucose and lactate.

Amperometric methods are utilized in most skin-integrated metabolic sensors owing to their intrinsic sensitivity, selectivity, and facile miniaturization. High sensitivity, conformal integration with targeted tissues are critical for detecting most analytes of interest on account of their low concentrations in these biofluids, which are also capable of preventing irritation and sample contamination. Surface micro-structured electrodes, which commonly exhibit higher catalytic performance in comparison with bulk structures, are leveraged to obtain high sensitivity for monitoring analytes at low concentrations by increasing the surface area, thereby augmenting the loading of reagents and establishing the stable transfer of electrical signals from reagents. Owing to unique physical and electrochemical properties, especially fast electron transfer and superior electrocatalytic activity, graphene can enhance the sensitivity of sensors. However, there is generally a lack of selectively without specific receptors such as enzymes, antibodies or DNA, which specifically bind to target analytes. Therefore, the reliance on labile biological receptors is the primary obstacle to robust biochemical sensors, which deteriorates if exposed to temperatures, pressures, or humidity levels out of the narrow range, or if stored with/without certain chemical species. For these sensors, robust operations are crucial in a relatively uncontrolled environment (i.e., time varying ambient, skin temperatures, oxygen levels, humidity and interfering chemistries, etc.). Thus, to minimize degradation and to promote sensor stability, incorporating multiple biochemical sensors onto a single platform and stabilizers (such as polyelectrolytes and polyols) are employed, whereas the lifetimes of sensors exists (Ray et al., 2018). Herein, graphene-based sensors for glucose monitoring in metabolites are highlighted.

**Figure 5:**
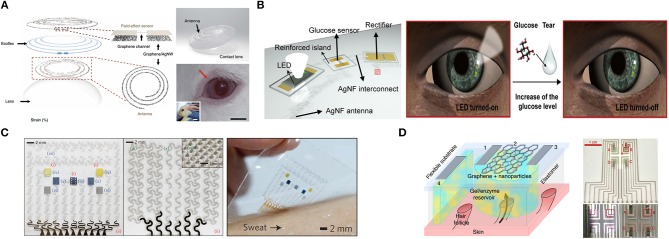
Graphene-based metabolic sensors. **(A)** Schematic of the wearable contact lens sensor and photograph of integrated onto the eyes of a live rabbit. Adapted with permission from Kim et al. (2017). **(B)** Schematic of the soft, smart contact lens and operation of this contact lens. Adapted with permission from Park J. et al. (2018). **(C)** Optical image of the electrochemical sensor array (left), therapeutic array (middle) and the diabetes patch laminated on human skin with perspiration (right). Adapted with permission from Lee et al. (2016). **(D)** Schematic of generic individual miniature pixel (left) and photographs of array realization (right). Adapted with permission from Lipani et al. (2018).

Tears also contain various biomarkers such as glucose, cholesterol, sodium ions, and potassium ions, which can be collected in the contact lens by thoroughly natural means, such as normal secretion and blinking (Farandos et al., 2015). As previously reported, the contact lens sensors can only monitor a single analyte at a time and employ opaque, brittle components as the electronic device, which could obstruct the wearer's view and potentially harm the eye. Moreover, signal measurements with costly and bulky equipment from the contact lens sensors could hinder the wearer in physical activities. Kim et al. (2017) developed a transparent (>91%), stretchable (~25%) and multifunctional wireless contact lens sensor that could monitor the glucose in tear fluid and intraocular pressure simultaneously without crosstalk, by utilizing the RLC circuit. A graphene-silver nanowire hybrid structure served as stretchable, transparent electrodes, resistors and antenna due to its enhanced electrical, mechanical properties without sacrificing transparency. A field-effect transistor (FET) mainly consisted of graphene/glucose oxidase as a channel and graphene-silver nanowire hybrid as a source/drain of electrodes, which responded to glucose in tears. Changes in capacitor that contains silicone elastomer as a dielectric layer and antenna could shift the resonance frequency at ocular hypertension (Figure 5A). After that, Park J. et al. (2018) proposed a soft, smart contact lens for real-time, wireless operation *in-vivo* tests to monitor the glucose concentration in tears with sensing results displayed simultaneously. The main functional devices including the rectifier circuit, a glucose sensor and light-emitting diode (LED) pixel were fixed on the reinforced islands of a hybrid substrate, while the stretchable, transparent antenna and interconnect electrodes made by silver nanofibers were located on elastic regions. The transparent and stretchable antenna with a rectifier droves the LED to display real-time sensing results wirelessly. Glucose oxidase (GOD) and catalase (CAT) was immobilized on the graphene surface with a pyrene linker by the p-p stacking interaction for glucose sensing. Finally, a live rabbit was utilized to tests *in-vivo* to prove its reliable operation without obvious adverse effects, which demonstrated a potential of the smart contact lenses for non-invasive tear-based health monitoring (Figure 5B).

Sweat has abundant analytes of interest such as glucose. An early example (Lee et al., 2015) proposed a graphene-functionalized stretchable device with transcutaneous drug delivery for sweat-based glucose monitoring and diabetes management. The device employed a soft, stretchable silicone membrane as a substrate and glucose oxidase/Au-doped graphene/Au mesh electrodes as a sensing element. These electrodes increased the surface area, improved electrochemical activity and established efficient electronic routes between the enzyme and the electrode surface, while maintaining the intrinsic softness of graphene. The device could detect glucose concentrations ranging from 10 μM and 0.7 mM in human perspiration with a high sensitivity and electrochemical performance under mechanical deformation (up to a 30% strain). The combined operation of the glucose sensor with other sensors including a potentiometric polyaniline-based pH sensor, a temperature sensor and a humidity sensor (for the pH, temperature calibration of the glucose sensor and monitoring the relative humidity to start glucose sensing, respectively) demonstrated robust performance in glucose sensing. Changes in the sweat glucose concentration are strongly correlated with those in blood with time lags. High glucose concentration detection could activate the embedded heaters to dissolve phase-change material (PCM) and bioresorbable microneedles deliver Metformin transcutaneously, which achieved a closed-loop for treatment of diabetes and showed immense potential for the treatment of chronic diseases (Figure 5C). For cases where there is little or no perspiration, sweat-inducing drugs including acetylcholine, pilocarpine, bethanechol, methacholine, and carbachol, can be employed by iontophoresis to induce a partial sweat response for subsequent sensing (Li et al., 2018). Additionally, different sweating rates, volumes as well as the pH of sweat could also affect the measurement.

ISF has been commonly extracted from skin using reverse iontophoresis (RI) via electrosmosis, which causes an ion flow of ISF from within the skin toward the cathode on the skin surface when a small electric field is applied across the skin. As a result, glucose within ISF can be detected and qualified. However, non-invasive, ISF-based continuous glucose monitoring devices commonly require finger-stick calibration after a few days because glucose is extracted randomly, varying across a relatively large area of skin (>3 cm^2^) and suffers substantial dilution before quantification. In fact, most of the electro-osmotic flow during iontophoresis follows low-resistance, prior pathways with hair follicles. Thus, Lipani et al. (2018) proposed a path-selective, non-invasive, transdermal hydrogel reservoir-based ISF-glucose monitoring pixel array platform. Glucose was extracted from individual, privileged follicular pathways across the skin and then detected via the pixels of the array. The miniaturized individual pixel array comprised an enzyme-encasing gel and CVD graphene decorating platinum nanoparticles on a RI to extract ISF. Graphene was desirable as it could be patterned and integrated on flexible substrates. A 2 × 2-pixel array was fabricated on a flexible substrate for mammalian skin *ex vivo* and critical performance of the monitor was evaluated and demonstrated. *In-vivo* continuous monitoring of glucose in ISF on healthy human subjects demonstrated the ability to continuously track glucose for 6 h with an applied current density of 1 or 2 mA.cm^−2^. This approach could assure that the ISF-glucose monitoring is not subject to inter- or intra-individual fluctuations in skin characteristics compared with the actual blood glucose concentrations, which paved a way to calibration-free non-invasive glucose monitoring (Figure 5D).

##### Electrolytes

Electrolytic sensors, which are mainly based on potentiometry, can also provide useful health information as discussed previously. A classical potentiometric sensor has an ion-selective electrode (ISE) and a reference electrode, in which the ISE potential is proportional to the ion activity complying with the Nernst equation and the reference electrodes (commonly Ag/AgCl) potential is independent of composition. Accordingly, ion activity is proportional to the potential difference between these two electrodes, which is almost the ion concentration. Wearable electrolytic sensors commonly leverage solid-state polyvinyl chloride (PVC)-based ion selective membranes rather than pH-sensitive conducting polymer membrane for pH sensing, in which the ion selective membrane contains an ionophore, ionic additives and a plasticizer for selectivity, charge transport and flexibility. The pH sensitive conducting polymers, such as polyaniline and polypyrrole, etc., have been employed by direct electrodeposition or solution casting onto noble metals or carbon electrodes in conducting polymer-based pH sensors. Signal stability is crucial for potentiometric sensors since tiny drifts in voltage lead to significant errors in the ion concentration. In addition, colorimetric analysis is an alternative for electrolyte detection with no need for a power supply (Li et al., 2018).

Wearable electrolytic sensors with the employment of varied graphene-based structures, such as sheets, flakes, 3D porous, field-effect transistors and nanocomposites, are capable of continuous monitoring of pH (Xuan et al., 2018a; Dang et al., 2019), potassium (He et al., 2017; Yuan et al., 2019), sodium (Ruecha et al., 2017) and chloride (Tseng et al., 2018), etc. The presence of graphene increases the hydrophobicity of the electrode surface, provides a high surface area, excellent conductivity, electrocatalytic activity and accelerates the electron transportation during ion exchange between an electrode and the solution.

##### Volatile biomarker gases

**Figure 6:**
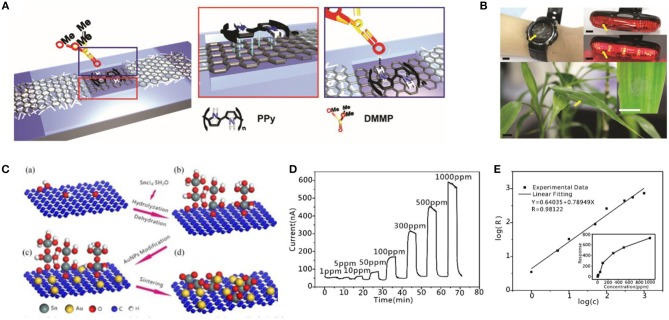
Structure diagram and its test diagram of graphene-based gas sensor. **(A)** Schematic illustrations of material coating on graphene channel. (Red box) π-π stacking interactions between graphene and PPy. (Blue box) hydrogen bonding between PPy and DMMP. **(B)** Photos of graphene gas sensors integrated with antenna fabricated or transferred onto various substrates. Adapted with permission from Park et al. (2016b). **(C)** Schematic illustration of the formation process of the Au/SnO_2_/RGO nanocomposites. **(D)** Responses of the Au/SnO_2_/RGO nanocomposites to ethanol. **(E)** Dilogarithm fit curve of the response (R) to the concentration (C). Adapted with permission from Meng et al. (2018).

Despite the metabolites and electrolytes detection, the volatile biomarker gases also contain vital information. For example, detecting the ammonia from human breath gases can diagnose helicobacter pylori infections in the stomach; measuring exhaled NO can diagnose asthma (Tricoli et al., 2017; Xu et al., 2018a). Nearly 2600 volatile organic compounds (VOCs) from breath, skin, urine and blood have been found to be vital auxiliary diagnostic references. Therefore, clinical diagnostics with the help of the precise measurement of VOCs yielded by human metabolism is an emerging approach. However, the key obstacle for accurate detection of VOCs is that extreme high selectivity and sensitivity of the target gases, blend with thousands of other gases and only a few particles per billion gas particles can be detected. In addition, volatile biomarker gas sensors should have high stability, reproducible responses, and fast recovery time. Based on the above requirements, graphene and its derivate display a potential candidate for application of the VOC sensors, which possess a large surface-to-volume ratio. These characteristics mean that graphene has a high sensitivity in detecting gas particles. Recently, there are enormous graphene-based biochemical sensors detecting dimethyl methylphosphonate (DMMP) (Park et al., 2016b), ethanol (Meng et al., 2018; Thu et al., 2018), NO_2_, SO_2_ (Cui et al., 2018) and tumor markers (Barash et al., 2015). Park et al. (2016b) presented an innovate DMMP gas sensor with high flexibility and transparency, which was fabricated by drop-coating polypyrrole onto graphene. This gas sensor exhibited excellent strain ability (up to 20%) and outstanding selectivity regardless of acetone, methanol, water as well as tetradecane. Concerning the ethanol gas, Thu et al. (2018) proposed a high-performance ethanol gas sensor, which was assembled of Fe_3_O_4_ and rGO. This gas sensor maintained a great selectivity of ethanol that the response of 100 ppm ethanol was 9.5, much higher than that of NH_3_, H_2_, and CO. It also had a fast response time, that the response (90%) time of the sensor was < 5s at 400°-500°C (Figures 6A,B). In addition, Meng et al. (2018) proposed a one-step method that fabricated ethanol gas sensor utilizing Au/SnO_2_ and rGO. This ethanol sensor exhibited a wide linear range (1–1,000 ppm) and high reproducibility (Figures 6C–E).

#### Environmental and Other Biochemical Applications

##### Gases

**Figure 7:**
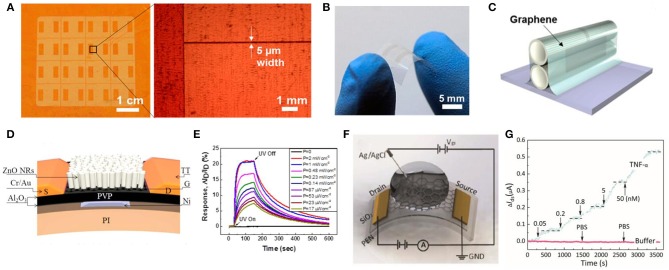
Schematic diagram and its test diagram of graphene-based environment signal sensor. **(A)** Optical microscopic images of patterned graphene on a Cu foil. **(B)** Photograph of a fabricated all-graphene gas sensor on a PI substrate. **(C)** Schematic for the patterned all-graphene sensor attached on ball pen leads. Adapted with permission from Kim et al. (2015). **(D)** Cross-sectional schematic of the hybrid FET photodetector on a flexible polyimide substrate. **(E)** Time-dependent responses of the flexible ZnO NR/Gr hybrid FET photodetector measured as a function of UV intensities at a fixed VG of 0 V and VD of 1 V. Adapted with permission from Dang et al. (2015). **(F)** Illustration of an electrolyte-gated flexible graphene field-effect transistor. **(G)** Real-time monitoring of changes in the TNF-α concentration. Adapted with permission from Hao et al. (2018).

Despite the flexible graphene-based wearable sensors for human health status, graphene and its derivatives have important applications in surrounding environment monitoring, which may be threatening to human health. It can be used in fabricating gas sensors to detect enormous hazardous gases such as NO_2_, NH_3_ and volatile gases, and also for ultraviolet (UV) rays. This kind of reaction, particularly the doping process, would induce the change of the electrical resistance of GO that can be detected by electrical methods. Su and Shieh (2014) proposed an innovative flexible NO_2_ gas sensor via a layer-by-layer (LBL) method that clings GO to a gold electrode. This gas sensor exhibited excellent flexibility of a 30° angle bent with 4% deviation and extreme sensitivity with the minimum detectable trace at 5 ppm with a mixture of 200 ppm of NH_3_. Furthermore, it showed outstanding long-term stability which maintained 86% of the initial performance by exposing 5 ppm of NO_2_ for 43 days. Yang et al. (2012) developed a flexible gas sensor with CVD-grown graphene as the active layer and a paper as the substrate. In this case, this gas sensor exhibited excellent flexibility that remained at 32–39% response when it was exposed to 200 ppm of NO_2_ with a strain of 0.5%. In view of transparency, Kim et al. (2015) innovated a flexible and transparent gas sensor based on an all-graphene structure (Figures 7A–C).

##### Light

Except for the detection of environmental chemical signals, a graphene-based sensor is also applied in environmental physical signals measurement, among which light signals are of vital importance. Considering the health of human skin, UV sensors exhibit a vital role in detecting the degree of UV intensity on the skin. Among the active materials for measuring UV, two-dimensional graphene is one of the potential candidates, displaying outstanding flexibility and high mobility at room temperature. Based on the ZnO nanorods (NRs) and graphene, Dang et al. (2015) proposed a flexible UV field effect transistor which showed high photoconductive gain (8.3 × 10^6^) under a gate bias of 5 V, high flexibility of a bend of 12 mm without performance degradation and high stability with UV response after 10,000 bend cycles (Figures 7D,E). Concerning the cost and high response, Wang et al. (2012) proposed a flexible UV sensor utilizing the rGO and hydrangea-like ZnO. This UV sensor exhibited a high photoresponse current (~1 μA), which was 700 times that of the ZnO UV sensor and a high on/off ratio (116/16), which increased one order of a magnitude compared with the original ZnO sensor.

##### Heavy metals

In addition, adhibitions of graphene are summarized in monitoring toxic heavy metal ions containing Cd^2+^, Hg^2+^, and Pb^2+^. Based on electrospinning, Yuan et al. (2015) proposed light-emitting nanofibrous films including conjugated microporous polymers (CMPs)/polylactic acid (PLA). The innovative chemical sensor showed high porosity, excellent flexibility and high surface-area-to-volume ratio which enhanced the ability to detect nitroaromatic, oxidizing heavy metal ions and so on. An et al. (2013) proposed a toxic Hg^2+^ sensor with liquid-gate FET-type and transparent graphene, which displayed high flexibility and specificity of Hg^2+^ despite other chemical substances.

##### Others

According to the above information, graphene and its derivatives exhibit outstanding characteristics such as excellent mechanical flexibility, electrical properties and long-term stability in measurement of vital human signals including bioelectrical signals, kinematic signals, and temperature signals. Except for the measurement of signals mentioned above, graphene-based biochemical sensors also occupy a small place in detecting biochemical signals such as dopamine (Tang et al., 2015; Raj et al., 2017), bacteria (Mannoor et al., 2012), acetyl choline (ACH) (Hess et al., 2014), ascorbic acid (Liu C. et al., 2016), medicine (Narang et al., 2016), cocaine (Hashemi et al., 2017), and tumor markers (Kwon et al., 2012; Azzouzi et al., 2015; Hao et al., 2018), which also have great significance in human health. For instance, Tang et al. (2015) proposed a graphene-modified acupuncture needle by incorporating graphene oxide and traditional needles, which showed an excellent sensitivity with a limit measurement of 0.24 μM, selectivity of dopamine and pH dependence (range from 2.0 to 10.0). As for detection of bacteria, in an early study, Mannoor et al. (2012) proposed a fully biointerfaced sensor for detection of bacteria from human respiration and saliva, which was fabricated by the self-assembly of antimicrobial peptides. This bacteria sensor exhibited single level detection and high sensitivity. Concerning the measurement of ACH, Chauhan et al. (2017) proposed a novel biochemical sensor combing reduced graphene oxide and enzyme acetylcholinesterase. In this case, this acetylcholine detection sensor displayed high electrical properties, particular selectivity, wide linear range (4.0 nM-800 μM) and a fast response time (< 4 s). With regards to the detection of cocaine, Hashemi et al. (2017) presented an innovative label free aptasensor employing magnetic reduced graphene oxide, polyaniline and gold nanoparticle, which exhibited a linear response to cocaine within 0.09 to 85 nM and high sensitivity (detection limit of 0.029 nM). In addition, there has been some exploratory work on graphene in detecting tumor cells. For instance, Azzouzi et al. (2015) combined reduced graphene oxide with _L_-lactate dehydrogenase to fabricate a particular sensor for _L_-lactate; Hao et al. (2018) proposed a graphene-based field effect transistor for the detection of cytokine biomarkers from human bodily fluids (Figures 7F,G).

### Invasive Sensors

While non-invasive wearable sensors are promising in human health monitoring, they do not have the ability to obtain data on the entire complexity of organ systems and long-term monitoring biological events continuously. Invasive sensors, which are close to the target organs or tissues, significantly increase the sensing accuracy and the curative effect in comparison to non-invasive counterparts. Thus, it is attracting a huge surge of interest in the monitoring, diagnosis, treatment, and management of diseases, which shows its potential in medical application (Eckert et al., 2013). As described previously, challenges including biocompatibility, biofouling, biodegradability, power supply, device minimization, integration, durability and lifetime, also exist in designing invasive sensors (Narayan and Verma, 2016; Gray et al., 2018). Although a large amount of work on implantable sensors for health monitoring has been done, graphene-based invasive sensors have been developed in limited aspects, such as neural recording and stimulation (Blaschke et al., 2017), glucose monitoring (Pu et al., 2018), cardiac monitoring (Chen et al., 2013) and EMG signals recording (Kim et al., 2016), which to date mainly focus on neural implants. The feasibility of graphene-based implants has been demonstrated *in vivo* in physiological systems, including the nervous system, cardiovascular system, digestive system and motional system.

#### Implants for Nervous System

Active neural implants that stimulate and/or record the electrical activity of the nervous system, can highlight the prospects for the clinical interventions and treatments of various diseases, such as Parkinson's disease, epilepsy, retinitis pigmentosa, pain or even psychiatric conditions (Kostarelos et al., 2017; Kireev et al., 2018). Moreover, brain-machine interfaces with neural implants allow for direct communication between the brain and machines (Choi J. et al., 2018). Although conventional non-invasive electrodes are capable of recording EEG signals (slow rhythms, 5–300 μV, < 100 Hz) from single or multiple sites on the scalp, the spatial resolution and SNR are undesired due to the filtering of different media, such as the skull and cerebral spinal fluid, which may not provide sufficient information to decode nerve signals. As an alternative, electrocorticography (ECoG) (medium rhythms, 0.01–5 mV, < 200 Hz) can achieve better spatial and time resolution and high SNR in an invasive way, by placing electrode arrays directly on the intracranial cortex. Penetrating electrodes are also utilized to record local field potentials (< 1 mV, < 200 Hz) and action potentials (ca. 500 μV, 0.1–7 kHz) (Fattahi et al., 2014). In order to improve the spatiotemporal resolution, microelectrode arrays (MEAs) with electrode diameters in tens of micrometers and electrode-to-electrode separation down to dozens of microns have been employed (Hebert et al., 2017). As with any implant, biocompatibility and non-immune responses are fundamental for electrodes. In addition, high flexibility (reaching conformability and stretchability) or Young modulus matching are crucial to minimize the movement within the soft tissue and to avoid shear-induced inflammation as the body moves, which cannot be achieved when utilizing rigid electrodes, such as silicon or noble metals. Furthermore, biochemical stability and electrical properties are also critical, indicating that the conductivity of electrodes must be high enough to enable safe stimulation or efficient recording signals in slightly salty, gel-like, and 37°C environments. Additionally, as the impedance and noise of the electrode are inversely proportional to electrode size, a trade-off is required between spatial resolution and SNR (Blaschke et al., 2017).

With the combination of extraordinary conductivity, electrochemical stability, flexibility, mechanical conformability and transparency, graphene is almost perfect in addressing many current challenges in neural interface design, where very few conductive polymers can claim all these features. Moreover, optical transparency of graphene is favorable for the study of neural networks and cortical features, where optogenetics, and calcium imaging at the same site can render complementary information (Kuzum et al., 2014; Park et al., 2014; Lu et al., 2018). Therefore, graphene microelectrode arrays (GMEAs) and graphene field effect transistors (GFETs) have been widely utilized for neural stimulation, recording and local preamplification (Thunemann et al., 2018). High surface-to-volume ratio makes graphene sensitive to charges at its surface. Furthermore, high transconductance and low intrinsic noise of GFETs, which require directly or extremely close to the electrode site, render capabilities in high SNR ratios recording with preamplification. As a consequence, the sensitivity to external noise is minimized.

Several structural innovations have been exploited in graphene-based materials for GMEAs and GFETs. For instance, wet-spun rGO fibers have been developed as free-standing penetrating electrodes in an early study (Apollo et al., 2015). Futhermore, highly crumpled all-carbon transistors with graphene channels and hybrid graphene/carbon nanotube electrodes have been achieved *in-vivo* recording of brain activity, with high sensitivity and substantially improved spatial resolution through aggressive in-plane compression (Yang L. et al., 2016). In addition, platinum nanoparticles (PtNPs) electrodeposited on monolayer graphene have been developed to overcome the quantum capacitance limitation and the lack of Faradaic reaction for the graphene electrodes (Du et al., 2018).

**Figure 8:**
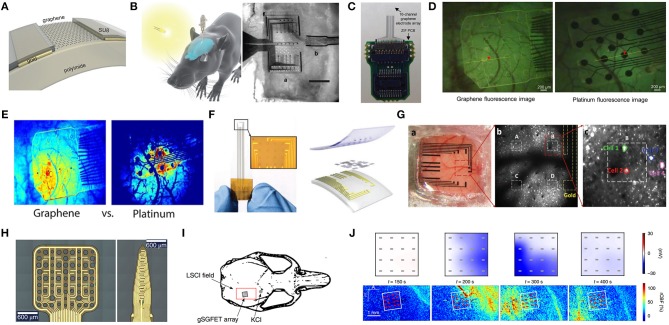
Graphene-based implants for nervous system. **(A)** Schematic of cross section of a graphene transistor. **(B)** Schematic of the implant placed on the surface of the rat's brain (left) and microscope image of a MEA with Pt electrodes and the graphene device next to it (right). Adapted with permission from Blaschke et al. (2017). **(C)** Graphene microECoG array with 16 transparent electrode sites and a ZIF PCB connector. **(D)** Visualization of the fluorescent neural response after stimulation with graphene electrodes (left) and platinum electrodes (right). **(E)** Visualization of the intensity of neural response to electrical stimulation with graphene electrode array and the same platinum electrode array. Adapted with permission from Park D. W. et al. (2018). **(F)** Photograph and Schematic of the array. **(G)** The PtNP/graphene electrode array placed on the cortex (left), two-photon microscope for cell bodies detection (middle) and image of multiple cells (right). Adapted with permission from Lu et al. (2018). **(H)** Optical microscope images of the active area of a 4 × 4 gSGFET array and a 15-channel intracortical array. **(I)** Schematic of a rat skull depicting the LSCI field of view and the position of the gSGFET array. **(J)** Electrical recordings and optical imaging were performed directly on the cortical surface. Color maps represent the spatial value of the extracellular voltage as measured by the gSGFET array and the rCBF at a given set of times after the induction of a CSD event. Adapted with permission from Masvidal-Codina et al. (2018).

Furthermore, operating *in-vivo* was recently performed (Liu T.C. et al., 2016; Park et al., 2017; Du et al., 2018; Lu et al., 2018). One study has employed flexible arrays of graphene solution-gated field-effect transistors to record brain activity *in vivo*, which shows a SNR of up to 72 compared to classical metal Pt electrodes of similar sizes (Blaschke et al., 2017). These graphene transistors have advantages such as intrinsic signal amplification, the possibility for down-scaling and high-density integration, which can compete with state-of-the-art MEAs technologies. The biocompatibility of the graphene implants has also been confirmed without any significant changes of circularity or solidity at any of the time points tested, compared to naive rats or polyimide samples without graphene (Figures 8A,B). Furthermore, imaging spatiotemporal neural responses to electrical stimulation with minimal artifacts can help to better understand the mechanisms of electrical stimulation in neural tissue and allow for various studies, which cannot be accomplished with existing opaque neural electrodes. Therefore, several studies (Liu X. et al., 2018; Lu et al., 2018; Thunemann et al., 2018) have developed fully transparent graphene electrodes for electrical brain stimulation and simultaneous optical monitoring of the underlying neural tissues.

For example, Park D. W. et al. (2018) developed a transparent graphene neural electrode, implanted in GCaMP6f mice, with capabilities in electrical stimulation and optical full-field monitoring of the neural tissues concurrently. With the employment of these transparent electrodes, fluorescence imaging of neural activity with minimal image artifacts was carried out in different electrical stimulation parameters, which also showed that more efficient neural activation could be obtained with cathode leading stimulation compared to that of anode. These graphene electrodes showed potential in therapeutic electrical stimulation of the nervous systems (Figures 8C–E). Although transparent graphene electrodes could enable simultaneous electrical stimulation and optical monitoring, the high impedance of the graphene obstructed wide application. Lu et al. (2018) demonstrated that quantum capacitance is the reason for high impedance of graphene electrodes. By electrodepositing platinum nanoparticles (PtNPs) on monolayers, the impedance of the PtNPs/graphene electrodes were dramatically reduced without a decrease in transparency. By utilizing transgenic mouse models, concurrently cortical activity recording with optical imaging was available with the PtNPs/graphene electrodes, which rendered the possibilities in figuring out the cellular dynamics as well as brain-scale neural activity (Figures 8F,G).

Monitoring brain activities below 0.1 Hz, commonly known as infralow activity (ISA), is valuable for clinical diagnosis, prognosis and therapy in neurocritical care, which can indicate brain states, such as sleep, or a coma. Cortical spreading depression (CSD), a slowly propagating wave of near-complete depolarization of neurons and astrocytes followed by a period of electrical activity suppression, occurs at infralow frequencies in brain pathophysiology, which is usually provoked in persons suffering a stroke, brain injury, and migraines. In order to record ISA *in-vivo*, Masvidal-Codina et al. (2018) exploited graphene solution-gated field-effect transistors (gSGFETs) arrays for both the epicortical and intracortical mapping of CSD. The results showed that graphene transistors were superb in recording ISA with spatially resolved mapping and could record in a wide frequency bandwidth from an infralow frequency to the typical local field potential bandwidth. With the employment of gSGFETs and optical techniques, such as laser speckle contrast imaging, 2D maps of neurovascular coupling could be obtained, which were significant in for a deep understanding of the neurovascular coupling phenomena (Figures 8H–J).

#### Implants for Cardiovascular System

In cardiovascular system, oxygenated blood is pumped to the whole body by the heart through the network of blood vessels. Diseases or even life threats may occur due to heart failure or a change in blood. Therefore, monitoring the biomarkers in blood and heart diseases is significant.

The concentration of blood glucose is a critical parameter in blood; hence blood glucose monitoring is significant, especially in diabetics. The glucose concentration of the venous plasma is regarded as the gold standard for glucose measurement. Although conventional glucose self-monitoring devices based on single-use test strips has been widely applied to improve the life quality for diabetes patients, it still has limitations such as pain, failure in measuring at sleep, as well as problems in continuous monitoring. Consequently, continuous glucose monitoring (CGM) is considered to be an optimized approach to obtain the illness state of diabetics for management of diabetes and complications. As discussed previously, biofluids-based non-invasive painless wearable glucose sensors are capable of continuous monitoring, however, they are still less accurate compared to direct blood glucose monitoring. Therefore, in order to measure blood glucose continuously, implantable glucose sensors and microdialysis-type devices have been developed (Lee et al., 2018). Several commercial state-of-the-art CGM systems do exist however, containing a minimally invasive needle-type sensor to monitor the glucose in the ISF, as the glucose concentrations in the ISF are closely related to those in the blood, and most of them rely on enzyme-based electrochemical detection (Bobrowski and Schuhmann, 2018). The implantable glucose sensors are also accompanied by some difficulties, including a short lifetime, biofouling and poor biocompatibility.

**Figure 9:**
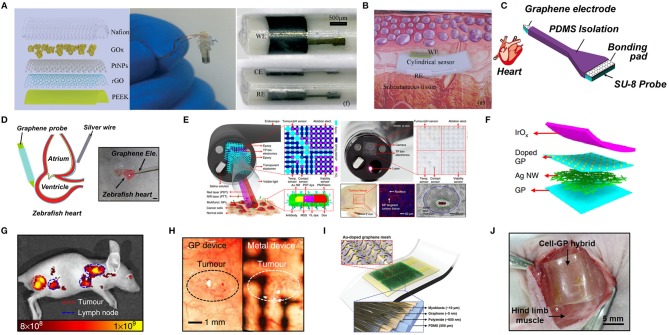
Other applications of invasive graphene-based sensors. **(A)** Schematic of the WE (left) and photos of the fabricated sensor (middle and right). **(B)** Schematic of implantable application of the flexible sensor in the subcutaneous tissue. Adapted with permission from Pu et al. (2018). **(C)** Schematic and optical micrographs of a flexible microprobe. **(D)** Schematic and actual view of the cardiac recording system for zebrafish. Adapted with permission from Chen et al. (2013). **(E)** Schematic illustrations and images of the multifunctional endoscope system based on transparent bioelectronic devices and theranostic nanoparticles. **(F)** Schematic illustration of the graphene hybrid in the exploded view. **(G)** Merged fluorescence image of the colon cancer on the mouse sub-dermis 6 h after intravenous injection of NPs. **(H)** Images of the tumor, captured by the camera of the endoscope through electronic devices (left: through transparent bioelectronic devices; right: through control metal devices). Adapted with permission from Lee et al. (2015). **(I)** Architecture of the stretchable and transparent cell-sheet-graphene hybrid. **(J)** Implantation of the cell-sheet-graphene hybrid onto target site of a nude mouse *in-vivo*. Adapted with permission from Kim et al. (2016).

Unfortunately, few invasive graphene-based glucose sensors have recently been reported. One recent study (Pu et al., 2018) proposed an inkjet printing based cylindrical flexible enzyme-electrode sensor for implantable CGM, to minimize signal drift and implement hypoglycemia detection. With the employment of a large surface area working electrode with 3D nanostructures consisting of graphene and platinum nanoparticles, the sensitivity was significantly enhanced with a detecting range of 0–570 mg.dL^−1^. An *in vivo* rat experiment showed that this sensor was promising in implantable CGM in subcutaneous tissue, which is comparable with commercial glucometers, even under hypoglycemic conditions (Figures 9A,B).

Heart failure is still a major public health problem, with a higher mortality rate than that of most cancers (Park et al., 2016a). As a primary organ of the cardiovascular system, heart activities can be recorded *in-vitro* and *in-vivo* for the early diagnosis and treatment of cardiovascular diseases. Until now, cardiac implanted devices including pacemakers and defibrillators, are capable of long-term sensing and pacing, diagnosis and treatment of rhythms and resynchronization, which can hardly be realized outside of the body (Freedman et al., 2017). Traditional pacing only activates the myocardium in leads, which may also face complications including lead failure, infection or tricuspid valve insufficiency (Bussooa et al., 2018). Leadless pacing is an alternative with a subcutaneous pocket and transvenous lead, which reduces complications (Merkel et al., 2017). Recently, electromechanical cardioplasty with an epicardial mesh has been employed to reconstruct cardiac tissue (Park et al., 2016a; Choi S. et al., 2018).

However, few studies are related to cardiac monitoring *in-vivo* with graphene-based electrodes. An early study (Chen et al., 2013) utilized steam plasma to treat the surface of a graphene-based flexible microprobe, which decreased the interfacial impedance, and thus high resolution and high SNR was obtained during neural and cardiac recording. The CVD prepared graphene electrode was in contact with a zebrafish heart to record the electrocardiographic signals. The signaling recording results exhibited that the QRS complex, *P* wave, and *T* wave were significantly increased in amplitude. The total noise of this microprobe was 4.2 μV_rms_ for hydrophilic the graphene-based sensor and 7.64 μV_rms_ for the hydrophobic graphene-based sensor (Figures 9C,D).

#### Implants for Digestive System

The digestive system supplies nutrients to the entire body, thus disorders of this system may lead to various associated diseases. the gastrointestinal tract is the largest structure of the digestive system and gastrointestinal diseases have become extremely common among the population (Yang N. et al., 2016). Minimally invasive surgical endoscopes with imaging and therapies are widely used to diagnose and treat gastrointestinal diseases. However, they lack spatial resolution in detecting and treating tiny cancers or other abnormalities. Thus, integrating electronic devices on the limited surface of cameras is required with transparent bioelectronics to avoid visual or light blockage. An early study (Lee et al., 2015) demonstrated a multifunctional endoscope system to diagnose and treat diseases like colon cancer, which contained graphene-based hybrid transparent electronic devices such as tumor, pH, viability, temperature sensors. Moreover, this closed-loop system contained radio frequency ablation as well as localized photo/chemotherapy, which could be utilized for colon cancer treatment *in-vivo*. This endoscope system enabled remarkable compatibility between the camera and the devices, accurate detection, delineation and fast targeted therapy (Figures 9E–H).

#### Implants for Locomotor System

The locomotor system provides the human body with the capability of movement through the muscular and skeletal systems. Accurate and continuous monitoring of EMG signals with instant feedback treatment is significant in diagnosing neuromuscular disorders, such as Duchenne muscular dystrophy and spinal muscular atrophy. Thus, one study (Kim et al., 2016) proposed a cell-sheet-graphene hybrid stretchable, transparent, implantable device with a high quality bio-interface to record EMG signals and stimulate muscles and nerves, which includes a sheet of C2C12 myoblast (~10 μm), Au-doped graphene mesh electrodes (~5 nm) with wrinkles, a polyimide (PI) membrane (~600 nm) and a PDMS substrate (500 μm). The cell-sheet-graphene hybrid with highly conductive Au doping graphene mesh electrodes was highly transparent, which could be employed to optically stimulate the modified muscle tissues. This device could be used *in vitro* for monitoring and stimulation of the C2C12 myoblasts. Moreover, *in-vivo* recordings of the EMG signals of hind-limb muscles in mice and electrical/optical stimulation of the implanted sites were applied without any immune reactions. This multifunctional device exhibited immense potential in soft bioelectronics (Figures 9I,J).

## Challenges and Future Outlook

The focus of human healthcare has shifted gradually from hospitals to communities (families, individuals). Tremendous effort has therefore been devoted toward sensors and devices for health monitoring. Due to its unique features, including chemical and physical properties, graphene is extremely attractive for flexible electronics and sensors. In this review, recent achievements in graphene-based sensors for human health monitoring, including both non-invasive flexible wearable sensors and invasive devices have been reviewed. The graphene-based sensors have been explored to measure a wide range of vital signs and biomarkers of the human body, which are highly promising in the foreseeable future for applications in healthcare, personalized/preventive medicine, disease treatment, human-machine interaction, as well as brain computer interfaces. Novel structures have been employed to improve performance, while their sensing mechanisms and technological innovations were also thoroughly discussed.

Non-invasive wearable sensors are more acceptable and desirable in healthcare applications, as they are less invasive, and reduce risks while maintaining their function and performance. Public attitudes toward wearable devices have changed from curiosity to clinical-grade healthcare (Rogers et al., 2019). However, there is still a long way to go before meeting the requirements of medical devices. With the progress of materials and manufacturing techniques, implantable medical devices are becoming increasingly attractive, because of their capability in long-term real-time accurate monitoring of the state of tissues, organs, system, while also further providing guidance/assistants/prognoses for diagnosis and therapeutics, which gradually replace traditional portable and wearable devices. However, for implantable devices, several challenges such as biocompatibility, biofouling, as well as power supply should be solved. Transient/biodegradable electronics show immense potential in implantable applications, which can be degraded in a manner of controlled triggers and/or self-triggering without secondary surgeries or risks of infection. Furthermore, the exciting thing is that the highly dispersed GO sheets can be biodegraded by myeloperoxidase which is derived from human neutrophils, which may be employed in biodegradable electronics for implants (Kurapati et al., 2015). In general, sensors for human health monitoring, whether being invasive or non-invasive sensors, can be considered as an “augmented sense,” which is an extension of human senses.

Considerable amounts of data will be generated with the development of sensor technologies and material science due to ubiquitous sensing ranging from the internet of things (IoT) to health care. Thus, statistical and computational methods, such as a range of machine learning techniques, can be utilized in data processing and effective information mining. Real-time data analytics capabilities are desired for robust data management (Paulovich et al., 2018). Ethical and moral issues in data collection, analysis and storage, particularly the data concerning personal health, must be properly resolved to protect personal privacy.

Although tremendous efforts have been devoted toward graphene-based sensors in recent years, a number of scientific and engineering challenges should be addressed before practical applications can proceed. For a start, human health risks such as the biocompatibility, biological toxicity, along with the environmental impact of graphene and its derivatives, need to be further assessed, especially in long-term *in-vivo* tests. Whole devices, with graphene as the core, are also required to be carefully checked. In addition, conformal, functional biotic/abiotic interfaces are crucial for robust sensing. Sensors on the epidermis and other organs with permeability to gases and moisture are desired. Moreover, high selectivity is required for multiple stimuli or ultra-low concentration biomarkers detection. The sensor may also be sensitive to stimuli other than the targeted stimulus to some extent, especially for integrated multifunctional sensors. For example, most sensors are affected by the environmental temperature floating. Crosstalk may exist in integrated multifunctional sensors, which can detect multi-signals simultaneously or separately. Long-term stability and mechanical durability are also demanded. Furthermore, integrated multifunctional sensors with feedback point-of-care therapy to construct a closed-loop system are significant in disease management. Power sources are essential for these devices, especially for implantable devices in long-term applications. Additionally, sensors combined with energy-harvesting technologies, such as triboelectrics nanogenerators (TENGs), photovoltaics, thermoelectrics, radio frequency (RF) and biofuel cells, are becoming a growing trend in the formation of self-powered systems (Liu et al., 2019). Finally, price and cost control are always an inevitable topic in commercialization. Therefore, cost-effective and facile fabrication methods with excellent uniformity should be developed for the large-volume high-throughput production of graphene and graphene-based sensors.

Each material has its unique advantages and limitations, and the requirements in different applications are also different, thus trade-offs are required. Although graphene provides a variety of distinctive characteristics in one, limitations also exist. First, a zero-gap structure of graphene results in the relatively low on/off ratio as FETs, which hinders its usability in biomedical applications. A possible way to open its bandgap is with functionalized organic molecules. Other attempts such as strain engineered lattice distortions, spintronics have also been explored. In addition, graphene is absent of selectivity toward target analytes of interest, owing to its excessive sensitivity to external stimuli. One possible approach to improve selectivity is to modify its surface with specific functional groups, bioreceptors or to cover it with a thin selective layer such as metal-organic frameworks (MOFs) (Tan et al., 2017). Furthermore, graphene has relatively low long-term stability induced by the moisture absorption and ultrathin nature. The solution may be to coat the surface with stable thin layer materials. Furthermore, the employment of graphene for functional devices in different applications requires a close integration with other functional materials; the intrinsic properties of graphene could be easily (usually negatively) impacted by these material integrations, device fabrication, and processing steps. Primary challenges including control, quality, scalability, and durability, should be resolved before commercially significant devices with graphene move forward.

## Author Contributions

HH collected all the references and wrote the review Part 1, 2, 3, and 4. NW and HW wrote the Part 2 and 3. SS edited the whole manuscript and sorted all the references. SW and HB managed the structure, provided constructive advices and suggestions. LS edited the whole paragraph and provided final revision.

### Conflict of Interest Statement

The authors declare that the research was conducted in the absence of any commercial or financial relationships that could be construed as a potential conflict of interest.
